# Is full adherence mandatory? Real-world outcomes of completing perioperative chemoimmunotherapy in resectable non-small cell lung cancer

**DOI:** 10.3389/fonc.2026.1837880

**Published:** 2026-05-28

**Authors:** Zhoujunyi Tian, Haoshuai Yang, Jin Zhang, Qiduo Yu, Deruo Liu, Meng Yang, Zhenrong Zhang, Chaoyang Liang

**Affiliations:** 1 Department of Thoracic Surgery, China-Japan Friendship Hospital, Beijing, China; 2 Department of Pulmonary and Critical Care Medicine, China-Japan Friendship Hospital, Beijing, China

**Keywords:** chemoimmunotherapy, non-adherence, non–small cell lung cancer, perioperative, recurrence-free survival

## Abstract

**Background:**

Neoadjuvant chemoimmunotherapy and adjuvant immunotherapy is a standard of care for resectable non-small cell lung cancer. However, high non-adherence rates challenge its implementation and benefits. We assessed the risks and long-term outcomes of completing this full regimen versus non-completion in a real-world setting.

**Methods:**

Patients with resectable non-small cell lung cancer at clinical stage IB-IIIB who received neoadjuvant chemoimmunotherapy followed by surgery were included. Patients were divided into a completed group (n=37), who received four cycles of perioperative chemoimmunotherapy and one year of adjuvant immunotherapy, and a non-completed group (n=127). Inverse Probability of Treatment Weighting was used to adjust baseline characteristics. Kaplan-Meier curves compared recurrence-free survival, overall survival, and cumulative hazard of adverse events. Cox regression models assessed predictors for recurrence and subgroup analyses were performed.

**Results:**

No significant differences were in recurrence-free survival (P = 0.85) or overall survival (P = 0.34) between the two groups. Positive pathological nodal status was an independent predictor of recurrence, while completion status was not (adjusted hazard ratio 1.14, 95% CI 0.24-5.50, P = 0.87). The completed group experienced a higher incidence of any-grade adverse events (AEs), but no significant increase in Grade 3/4 AEs. Subgroup analysis revealed a notable recurrence benefit from completion in patients who were aged <65 years and ECOG score 0.

**Conclusion:**

Completing perioperative chemoimmunotherapy protocol did not significantly increase the risk of severe AEs, but also failed to demonstrate a survival benefit. Prospective studies and extended follow-up duration were needed to verify the conclusions.

## Introduction

Lung cancer is the leading cause of cancer-related death worldwide. Non-small cell lung cancer (NSCLC) accounts for approximately 85% of all lung cancers (1). Approximately 30% of NSCLC patients are resectable at the time of diagnosis (2). However, 30% to 50% develop lung cancer-related recurrence and death after the surgery (3). Even with perioperative chemotherapy, the 5-year survival rate further increased by only approximately 5% (4). In the past few years, the emergence of immune checkpoint inhibitors (ICIs) has rapidly shifted the treatment paradigm for patients with resectable NSCLC. The checkmate 816 trial confirmed that neoadjuvant chemoimmunotherapy significantly increased the rate of pathological complete response (pCR), event-free survival (EFS), and ultimately translate into overall survival (OS) benefits compared to traditional neoadjuvant chemotherapy, which has established the position of neoadjuvant chemoimmunotherapy in the treatment of resectable NSCLC with no known EGFR mutations or ALK rearrangements (5). The checkmate 77T, AEGEAN and Keynote 671 trials established 4 cycles of neoadjuvant chemoimmunotherapy and one-year adjuvant immunotherapy after the surgery. They confirmed that this therapy modality could significantly increase the pCR rate and EFS compared to 4-cycle neoadjuvant chemotherapy. And Keynote 671 also reported OS benefit (6–8). On the basis of these crucial phase 3 trials, 4-cycle neoadjuvant chemoimmunotherapy with one-year adjuvant immunotherapy was recommended in the United States, the European Union, China and several other countries for eligible patients with resectable NSCLC (9–11).

However, it is hard to fully implement the perioperative treatment plan. Taking into account the toxicity of neoadjuvant chemoimmunotherapy and surgical cancellation, the Rationale-315 trial employed a more flexible 3 or 4-cycle neoadjuvant chemoimmunotherapy regimen (12). The Neotorch trial employed three cycles of neoadjuvant chemoimmunotherapy followed by one cycle of adjuvant chemoimmunotherapy and then continued with adjuvant immunotherapy, which ensured that patients received a total of 4 cycles of perioperative chemoimmunotherapy while allowing surgeons to determine the optimal cycles of neoadjuvant therapy and surgical timing (13). Even in the context of phase 3 clinical trials, the proportion of patients who fulfilled neoadjuvant chemoimmunotherapy was 74.4%-93.8%, while the proportion of those who fulfilled one-year adjuvant immunotherapy was 24%-48.2% (9–11). In clinical practice, we may encounter more resistance when treat patients according to the guidelines.

Based on the issues revealed by the above randomized controlled trials, as well as the remaining controversial issues such as the ideal duration for neoadjuvant chemoimmunotherapy and the necessity of adjuvant immunotherapy, we conducted this real-world study to assess the risks and benefits of completing perioperative immunotherapy, and to help us determine which subgroup of patients are more likely to benefit from the completed treatment.

## Patients and methods

### Ethics statement

This retrospective study was approved by the Institutional Review Board of China-Japan Friendship Hospital (ID: 2023-KY-151, date: July 5, 2023). This study was conducted in accordance with the principles of the Declaration of Helsinki. Informed consent of patients was waived given the retrospective setting.

### Patient selection

Data for patients with resectable stage IB-IIIB NSCLC [according to the American Joint Committee on Cancer (AJCC) 8th edition guidelines] at China-Japan Friendship Hospital between June 2019 and December 2024 were reviewed.

The inclusion criteria were as follows: (1) Aged 18–75 years; (2) No EGFR mutations or known ALK translocations; (3) Received neoadjuvant chemoimmunotherapy followed by surgery; (4) Cytology and histology were examined via bronchoscopy or aspiration biopsy prior to treatment; and (5) ECOG performance status 0-1.

The exclusion criteria were as follows: (1) Aged <18 or >75 years; (2) Receiving neoadjuvant immunotherapy alone or neoadjuvant chemotherapy alone; and (3) Patients whose objective of surgery was not radical resection, such as biopsy, wedge resection or segment resection; and (4) Incomplete follow-up data.

All patients underwent 18F-fluorodeoxyglucose positron emission tomography/computed tomography before treatment, or underwent enhanced chest and abdomen CT, cranial magnetic resonance imaging, and whole-body bone scintigraphy as alternatives. The treatment strategy for each patient was decided by a multidisciplinary team that included oncologists, surgeons, radiologists and pathologists. Flowchart of selection of study cohort was shown in **Supplementary Figure 1**.

Patients were divided into two groups based on whether they met the criteria of completing 4 cycles of perioperative chemoimmunotherapy and one year of postoperative single-agent adjuvant immunotherapy. Data on patients’ clinical and pathological characteristics, perioperative outcome, surgical difficulty and long-term outcomes were collected. Surgery delay refers to an interval of more than 6 weeks from the last neoadjuvant treatment to surgery. Surgical specimens, including the primary tumor and dissected lymph nodes, were evaluated by two senior pathologists. The pathological response was evaluated by calculating the average percentage of viable tumor cells. pCR is defined as the absence of residual living tumor cells in the primary tumor and dissected lymph nodes. Adverse events were graded according to the National Cancer Institute Common Terminology Criteria for Adverse Events (CTCAE) version 5.0.

### Inverse probability of treatment weighting

To minimize the bias between the two groups caused by patient baseline characteristics, we employed the inverse probability of treatment weighting method (IPTW) (14, 15). Propensity score was calculated using a multivariate logistic regression model, which included the following pre-treatment covariates: age, sex, clinical T stage, clinical N stage, tumor location, comorbidity, smoking history, ECOG performance-status score, pulmonary function indicators, pathological subtype, PD-L1 expression level, and lobe.

Covariate balance after IPTW was assessed using standardized mean differences (SMD). An absolute SMD <0.10 was considered indicative of adequate balance between treatment groups. SMDs before and after weighting were reported in **Supplementary Figure 2**. We also performed a sensitivity analysis using E-values as an alternative to IPTW (16). The E-value represents the minimum strength of association that an unmeasured confounder would need to have with both treatment and outcome to fully explain away the observed association. The result was shown in **Supplementary Figure 3**.

The weight of each patient was determined by the inverse probability of being assigned to either the completed group or not-completed group. IPTW-adjusted Kaplan-Meier curves with log-rank tests was used to compare recurrence-free survival (RFS) and overall survival (OS) between the two groups. And IPTW-adjusted cumulative hazard curves were used to compare treatment related adverse event. We also estimated the effect of completing perioperative immunotherapy on RFS using the IPTW-adjusted univariate and multivariate Cox regressions. Subgroup analysis was conducted using an IPTW-adjusted Cox regression model to explore which kind of patients might achieve better RFS from completing perioperative immunotherapy.

### Statistical analyses

Categorical variables were compared using Pearson’s χ^2^ test or Fisher’s exact test. Continuous variables that were normally distributed were evaluated using the Student’s t test, whereas variables that were not normally distributed were compared using the Mann-Whitney U test.

A two-sided P value < 0.05 was considered statistically significant. All statistical analyses were performed using Stata/SE version 14.0 for Windows (StataCorp, College Station, TX, USA) and R for Windows version 4.3.2 [R Core Team (2023). R: A Language and Environment for Statistical Computing. R Foundation for Statistical Computing, Vienna, Austria. https://www.R-project.org/].

## Result

### Patient selection and treatment completion patterns

A total of 164 patients were included, among who 37 (completed group) met the criteria of completing four cycles of perioperative chemoimmunotherapy and one year of postoperative single-agent adjuvant immunotherapy and 127 (not-completed group) did not (**Supplementary Figure 1**). The overview of treatments which patients received in the study was shown in the Sankey diagram (**Supplementary Figure 4**). Among the 37 patients in the completed group, 14 (37.8%) completed all four cycles entirely in the neoadjuvant period, while the remaining 23 (62.2%) completed three cycles in the neoadjuvant period and one cycle in the adjuvant period, followed by one year of adjuvant immunotherapy. In the not-completed group, 15 patients (11.8%) completed 4 cycles of chemoimmunotherapy in neoadjuvant therapy period but did not complete one year of adjuvant immunotherapy; 36 patients (28.3%) completed 4 cycles of perioperative chemoimmunotherapy but did not complete one year of adjuvant immunotherapy; 24 patients (18.9%) did not complete 4 cycles of chemoimmunotherapy but completed one year of adjuvant immunotherapy; 52 patients (40.9%) failed to complete either of these two treatments.

### Baseline characteristics and IPTW analysis

Patient clinical and pathological characteristics before and after IPTW are shown in **Table 1**. After IPTW adjustment, all pre-treatment covariates achieved adequate balance between the completed and not-completed groups. As shown in **Supplementary Figure 2**, most of pre-treatment covariates achieved adequate balance after IPTW, falling within the “well-balanced” zone.

**Table 1:** Patient clinical and pathological characteristics.

Variables	Before IPTW			After IPTW		
	Completed group (n=37)	Not-completed group (n=127)	P	Completed group (n=122)	Not-completed group (n=171)	P
Age						
Median (Q1,Q3),yr	63 (57,68)	64 (59,68)	0.50	64 (59,70)	65 (59,67)	0.52
Distribution, n (%)			0.46			0.21
<65 yr	23 (62.2)	68 (53.5)		70 (57.6)	84 (49.5)	
≥65 yr	14 (37.8)	59 (46.5)		52 (42.4)	86 (50.5)	
Gender, n (%)			0.52			1
Male	35 (94.6)	114 (89.8)		112 (92.1)	156 (91.5)	
Female	2 (5.4)	13 (10.2)		10 (7.9)	14 (8.5)	
BMI, mean ± SD	24.5 ± 3.5	24.4 ± 3.4	0.91	24.5 ± 3.5	24.1 ± 3.4	0.65
Smoking history, n (%)	30 (81.1)	108 (85.0)	0.75	99 (81.4)	142 (83.3)	0.79
Pathological Subtype, n (%)			0.92			0.63
Squamous carcinoma	30 (81.1)	100 (78.7)		95 (77.5)	138 (81)	
Adenocarcinoma	6 (16.2)	24 (18.9)		26 (21)	29 (16.9)	
Large cell carcinoma	1 (2.7)	3 (2.4)		2 (1.5)	4 (2.1)	
Pulmonary function						
FEV1/FVC (%), median (Q1,Q3)	72.8 (65.7,77.0)	72.9 (66.4,77.5)	0.80	71.9 (61.6,76.5)	72.3 (62.9,76.7)	0.88
FEV1%, median (Q1,Q3)	89.3 (79.8,99.9)	92.9 (76.8,103)	0.54	90.7 (78.8,100.8)	90.3 (73.3,102.7)	0.92
DLCO SB%, mean ± SD	78.2 ± 19.2	76.0 ± 15.3	0.62	79.2 ± 19.7	78.2 ± 16.0	0.86
Comorbidity, n (%)						
Hypertension	16 (43.2)	63 (49.6)	0.62	45 (36.8)	77 (44.9)	0.21
Diabetes	11 (29.7)	28 (22)	0.46	27 (22.1)	41 (23.8)	0.84
Cardiac disease	3 (8.1)	17 (13.4)	0.57	14 (11.8)	20 (11.8)	1
COPD	11 (29.7)	53 (41.7)	0.26	45 (36.7)	69 (40.3)	0.62
Other	3 (8.1)	14 (11)	0.77	15 (12.4)	17 (10.1)	0.67
ECOG performance-status score, n (%)			1			0.56
0	107 (84.3)	31 (83.8)		99 (80.9)	144 (84.2)	
1	20 (15.7)	6 (16.2)		23 (19.1)	27 (15.8)	
Tumor location, n (%)			0.73			0.69
Peripheral	8 (21.6)	33 (26.2)		32 (26.1)	40 (23.3)	
Central	29 (78.4)	93 (73.8)		90 (73.9)	131 (76.7)	
Lobe, n (%)			0.25			0.36
Right upper	9 (24.3)	32 (25.2)		31 (25.5)	41 (24)	
Right middle	1 (2.7)	9 (7.1)		2 (1.5)	10 (5.6)	
Right lower	9 (24.3)	31 (24.4)		22 (18.1)	38 (22.4)	
Left upper	13 (35.1)	24 (18.9)		36 (29.2)	45 (26.4)	
Left lower	5 (13.5)	31 (24.4)		31 (25.6)	37 (21.7)	
AJCC 8th clinical T stage, n (%)			0.28			0.14
T1	6 (16.2)	16 (12.6)		13 (11)	20 (11.9)	
T2	15 (40.5)	69 (54.3)		48 (39.7)	85 (49.9)	
T3	9 (24.3)	30 (23.6)		43 (35.6)	40 (23.4)	
T4	7 (18.9)	12 (9.4)		17 (13.7)	25 (14.8)	
AJCC 8th clinical N stage, n (%)			0.08			0.45
N0	8 (21.6)	53 (41.7)		34 (28.3)	60 (35.3)	
N1	8 (21.6)	23 (18.1)		23 (19.3)	29 (17.2)	
N2	21 (56.8)	51 (40.2)		64 (52.5)	81 (47.5)	
PD-L1 expression, n (%)			0.08			0.55
Unknown	27 (73)	110 (86.6)		97 (79.4)	146 (85.7)	
<1% of tumor cells	4 (10.8)	3 (2.4)		5 (4.5)	5 (2.9)	
1%-<50% of tumor cells	4 (10.8)	9 (7.1)		13 (10.6)	12 (7)	
≥50% of tumor cells	2 (5.4)	5 (3.9)		7 (5.5)	8 (4.4)	
Surgery delay, n (%)	10 (27)	28 (22)		29 (23.5)	35 (20.4)	0.63
AJCC 8th yp T stage, n (%)			0.63			0.73
T0	18 (48.6)	55 (43.3)		48 (39.5)	70 (41)	
T1	15 (40.5)	54 (42.5)		58 (47.9)	71 (41.5)	
T2	2 (5.4)	14 (11)		13 (10.4)	25 (14.6)	
T3	1 (2.7)	3 (2.4)		1 (1.2)	4 (2.1)	
T4	1 (2.7)	1 (0.8)		1 (1)	1 (0.7)	
AJCC 8th yp N stage, n (%)			0.57			0.04
N0	26 (70.3)	98 (77.2)		84 (69.3)	135 (79.3)	
N1	7 (18.9)	16 (12.6)		28 (22.9)	20 (11.8)	
N2	4 (10.8)	13 (10.2)		9 (7.8)	15 (8.9)	
pCR, n (%)	12 (32.4)	53 (41.7)	0.41	28 (23)	68 (39.6)	**0.004**

P values in bold are statistically significant.IPTW, inverse probability of treatment weighting; Q1, first percentile; Q3, third percentile; DLCO SB, Diffusing capacity of the lung for carbon monoxide, single−breath method; %, Its percentage of prediction value; FVC, Forced−vital capacity; FEV1, Forced expiratory volume in 1 s, FEV1/FVC (%), The percentage calculated by dividing FEV1 by FVC; SD, Standard deviation; COPD, Chronic obstructive pulmonary disease; ECOG, Eastern Cooperative Oncology Group; AJCC, American Joint Committee on Cancer; PD-L1, programmed death ligand 1; pCR, pathological complete response.

In the overall study population, the majority were male (n=149, 90.1%), had a smoking history (n=138, 84.1%), and had squamous cell carcinoma (n=130, 79.3%). After the IPTW, the proportion of ypN1 in the completed group was significantly higher than that in the non-completed group (22.9% vs 11.8%, p = 0.04), while the pCR rate was lower than that in the non-completed group (23.0% vs 39.6%, p = 0.004). The regimens for chemoimmunotherapy are shown in **Supplementary Table 1**. Paclitaxel plus platinum (81.1% of total) and tislelizumab (45.1% of total) were the most commonly used regimens for chemotherapy and immunotherapy respectively. Perioperative outcomes were shown in **Supplementary Table 2**. No significant difference was observed in surgical approach, surgical procedure, R0 rate, operation duration, intraoperative blood loss volume, chest tube duration, postoperative hospital stay and incidence of surgery complications between the two groups.

### Survival analysis and safety profile

**Figure 1:**
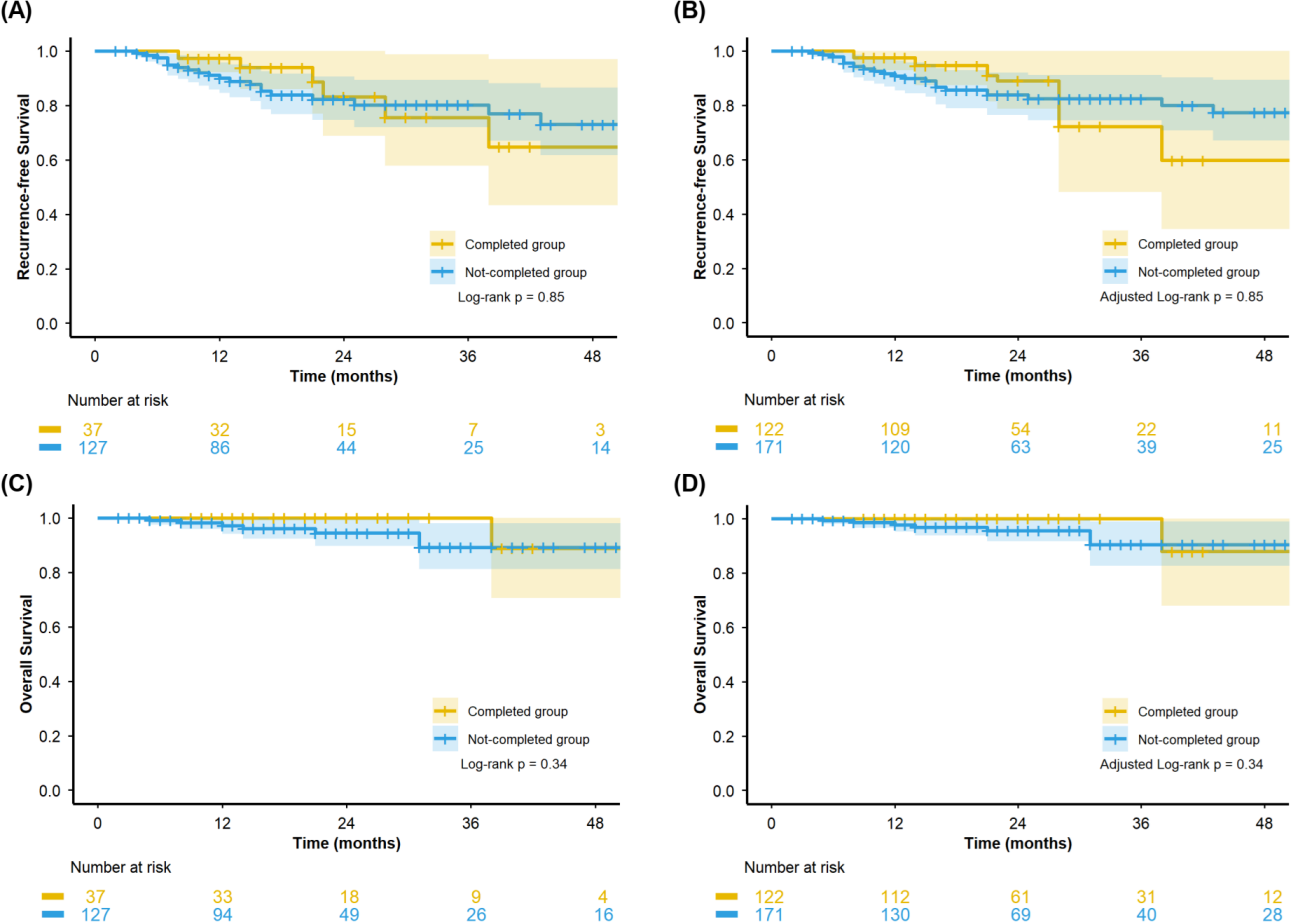
**(A)** Kaplan-Meier curve of recurrence-free survival (RFS) between completed group and not-completed group (log-rank P = 0.85); **(B)** IPTW (inverse probability of treatment weighting)-adjusted Kaplan-Meier curve of RFS between the two groups (adjusted log-rank P = 0.85); **(C)** Kaplan-Meier curve of overall survival (OS) between the two groups (log-rank P = 0.34); **(D)** IPTW-adjusted Kaplan-Meier curve of OS between the two groups (adjusted log-rank P = 0.34).

Recurrence-free survival and overall survival analysis was performed. The median follow-up duration was 20 months (95% confidence interval [CI] 17–24 months). Kaplan-Meier analyses revealed no significant differences in RFS (log-rank P = 0.85) (**Figure 1A**) and OS (log-rank P = 0.34) (**Figure 1C**) between the two groups. IPTW-adjusted Kaplan-Meier curves with log-rank tests consistently showed no significant differences in RFS (adjusted log-rank P = 0.85) (**Figure 1B**) and OS (adjusted log-rank P = 0.34) (**Figure 1D**).

**Figure 2:**
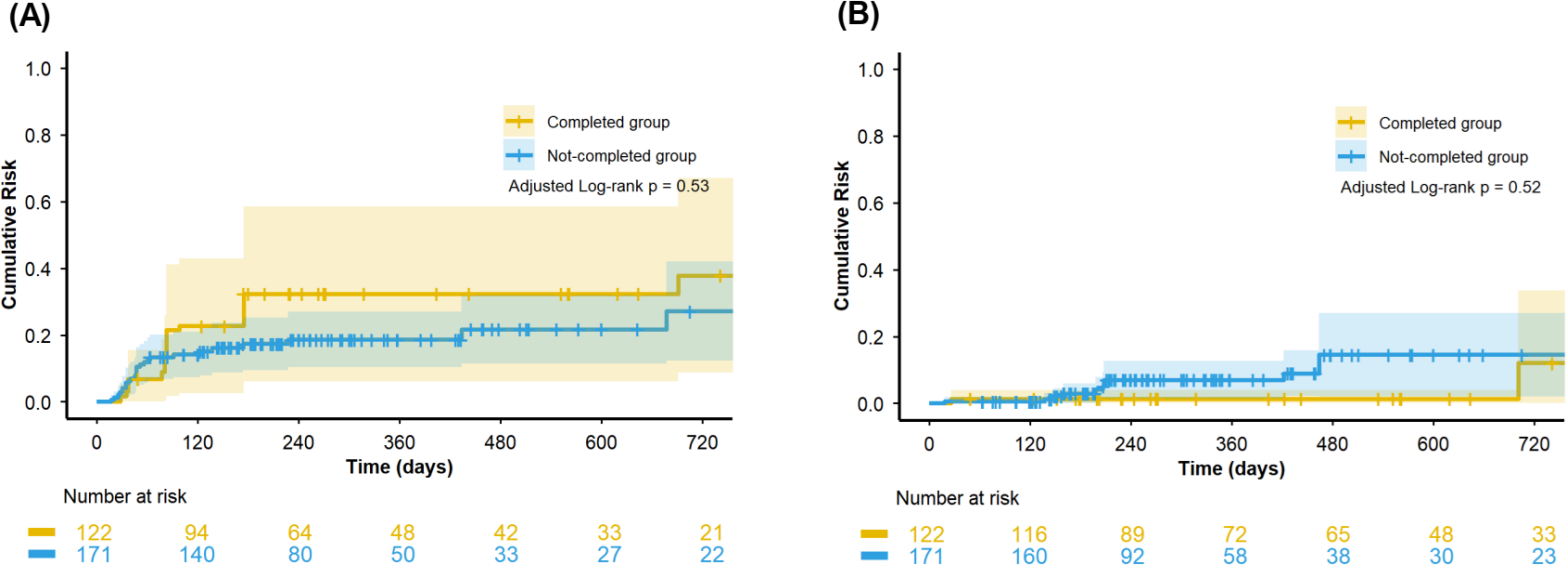
**(A)** IPTW (inverse probability of treatment weighting)-adjusted cumulative hazard curve of adverse events related to myelosuppression between the two groups (adjusted log-rank P = 0.53); **(B)** IPTW-adjusted cumulative hazard curve of immune-related adverse events between the two groups (adjusted log-rank P = 0.52).

Adverse events (AEs) incidence in the two groups after IPTW were shown in **Table 2**. Both in the overall treatment process and in the neoadjuvant period, the incidence of AE related to myelosuppression was significantly higher in the completed group. Only in the overall treatment process, the incidence of immune-related AE (irAE) was significantly higher in the completed group. However, AEs of grade 3 or 4 did not show a significant increase. The detailed information on AEs during the neoadjuvant and the adjuvant period can be found in **Supplementary Table 3** and **Supplementary Table 4** respectively. IPTW adjusted cumulative hazard curves revealed no significant difference in risk of grade3 or 4 AE related to myelosuppression (adjusted log-rank P = 0.53) (**Figure 2A**) and irAE (adjusted log-rank P = 0.52) (**Figure 2B**).

**Table 2:** Summary of adverse events.

Variable	Overall			Neoadjuvant period			Adjuvant period		
	Completed group (n=37)	Not-completed group (n=127)	P	Completed group (n=37)	Not-completed group (n=127)	P	Completed group (n=37)	Not-completed group (n=127)	P
Before IPTW									
AEs related to myelosuppression, n (%)									
Any grade	27 (73)	61 (48)	**0.013**	25 (67.6)	54 (42.5)	**0.013**	9 (24.3)	13 (10.2)	0.051
Grade 3 or 4	10 (27)	25 (19.7)	0.47	7 (18.9)	21 (16.5)	0.93	3 (8.1)	5 (3.9)	0.38
irAEs, n (%)									
Any grade	17 (45.9)	31 (24.4)	**0.02**	11 (29.7)	22 (17.3)	0.16	9 (24.3)	13 (10.2)	0.051
Grade 3 or 4	2 (5.4)	9 (7.1)	1	1 (2.7)	5 (3.9)	1	1 (2.7)	5 (3.9)	1
After IPTW									
AEs related to myelosuppression, n (%)									
Any grade	77 (70.7)	74 (46.7)	**<0.001**	65 (59.3)	67 (42.1)	**0.008**	34 (31.3)	18 (11.3)	**<0.001**
Grade 3 or 4	32 (29.2)	30 (18.8)	0.067	22 (20.1)	25 (16.1)	0.49	10 (9.1)	5 (3.4)	0.09
irAEs, n (%)									
Any grade	42 (38.3)	39 (24.4)	**0.021**	27 (25.1)	27 (17.2)	0.15	22 (20)	18 (11.6)	0.088
Grade 3 or 4	2 (2.1)	13 (8.5)	0.053	1 (1.1)	8 (5)	0.16	1 (1)	9 (5.9)	0.084

P values in bold are statistically significant. IPTW, inverse probability of treatment weighting; AEs, adverse events; irAEs, immune-related adverse events.

### Cox regression and subgroup analysis

IPTW-adjusted univariate and multivariate Cox regression analyses were performed to identify independent predictors of RFS (**Table 3**). Completion of perioperative chemoimmunotherapy was not an independent predictor of RFS [adjusted hazard ratio (aHR) 0.65, 95% confidence interval (CI) 0.31-1.39, P = 0.27]. Pathological nodal status emerged as strong predictor of recurrence: ypN2 (aHR 35.56, 95% CI 10.71-118.08, P < 0.001) was significantly associated with worse RFS compared with ypN0. Similarly, ypT3 (aHR 45.93, 95% CI 5.17-408.49, P < 0.001) and ypT4 (aHR 41.75, 95% CI 5.57-313.13, P < 0.001) were strongly associated with increased recurrence risk compared with ypT0. Female sex was associated with significantly better RFS (aHR 0.15, 95% CI 0.03-0.69, P = 0.02), while adenocarcinoma was associated with worse RFS compared with squamous cell carcinoma (aHR 4.15, 95% CI 1.41-12.19, P = 0.01).

**Table 3:** IPTW-adjusted Univariate and multivariate Cox regressions analyses for identifying independent factors of RFS.

Variable	Univariate regression			Multivariate regression		
	aHR	95% CI	P	aHR	95% CI	P
Completed perioperative therapy						
No	Ref			Ref		
Yes	1.12	0.63-2.00	0.69	0.65	0.31-1.39	0.27
Age						
<65 yr	Ref					
≥65 yr	0.67	0.37-1.21	0.19			
Gender						
Male	Ref			Ref		
Female	2.33	1.14-4.78	**0.02**	0.15	0.03-0.69	**0.02**
BMI	1.06	0.98-1.15	0.15			
Smoking history	0.55	0.28-1.08	0.08			
Pathological Subtype						
Squamous carcinoma	Ref			Ref		
Adenocarcinoma	2.82	1.52-5.24	**0.001**	4.15	1.41-12.19	**0.01**
Large cell carcinoma	NA		0.99	NA		0.99
Comorbidity						
Hypertension	1.21	0.67-2.18	0.52			
Diabetes	0.62	0.28-1.41	0.26			
Cardiac disease	0.24	0.04-1.41	0.11			
COPD	1.43	0.80-2.56	0.23			
Other	3.36	1.70-6.63	**<0.001**	2.01	0.73-5.56	0.18
Tumor location						
Peripheral	Ref					
Central	0.65	0.33-1.28	0.22			
AJCC 8th cT stage						
T1	Ref					
T2	1.91	0.62-5.94	0.26			
T3	0.51	0.13-2.03	0.34			
T4	1.07	0.29-3.88	0.92			
AJCC 8th cN stage						
N0	Ref			Ref		
N1	1.98	0.71-5.49	0.19	3.02	0.86-10.62	0.08
N2	2.11	1.00-4.45	**0.049**	1.75	0.66-4.63	0.26
Surgery delay						
No	Ref					
Yes	1.28	0.64-2.55	0.49			
Resection completeness						
R0	Ref					
R1/2	2.18	0.73-6.56	0.17			
AJCC 8th yp T stage						
T0	Ref			Ref		
T1	2.11	0.97-4.62	0.06	1.10	0.37-3.27	0.86
T2	2.49	1.02-6.10	**0.046**	1.57	0.40-6.16	0.52
T3	5.10	1.20-21.62	**0.03**	45.93	5.17-408.49	**<0.001**
T4	25.17	6.04-104.81	**<0.001**	41.75	5.57-313.13	**<0.001**
AJCC 8th yp N stage						
N0	Ref			Ref		
N1	3.05	1.55-6.00	**0.001**	2.72	1.00-7.39	0.05
N2	13.83	6.24-30.66	**<0.001**	35.56	10.71-118.08	**<0.001**
pCR						
No	Ref			Ref		
Yes	0.13	0.03-0.50	**0.003**	0.68	0.10-4.67	0.70

P values in bold are statistically significant. 95% CI, 95% confidence interval; aHR, adjusted hazard ratio; NA, not available, this occurs when the HR value is significantly greater than 1 or significantly less than 1, and the 95% CI crosses 1.

**Figure 3:**
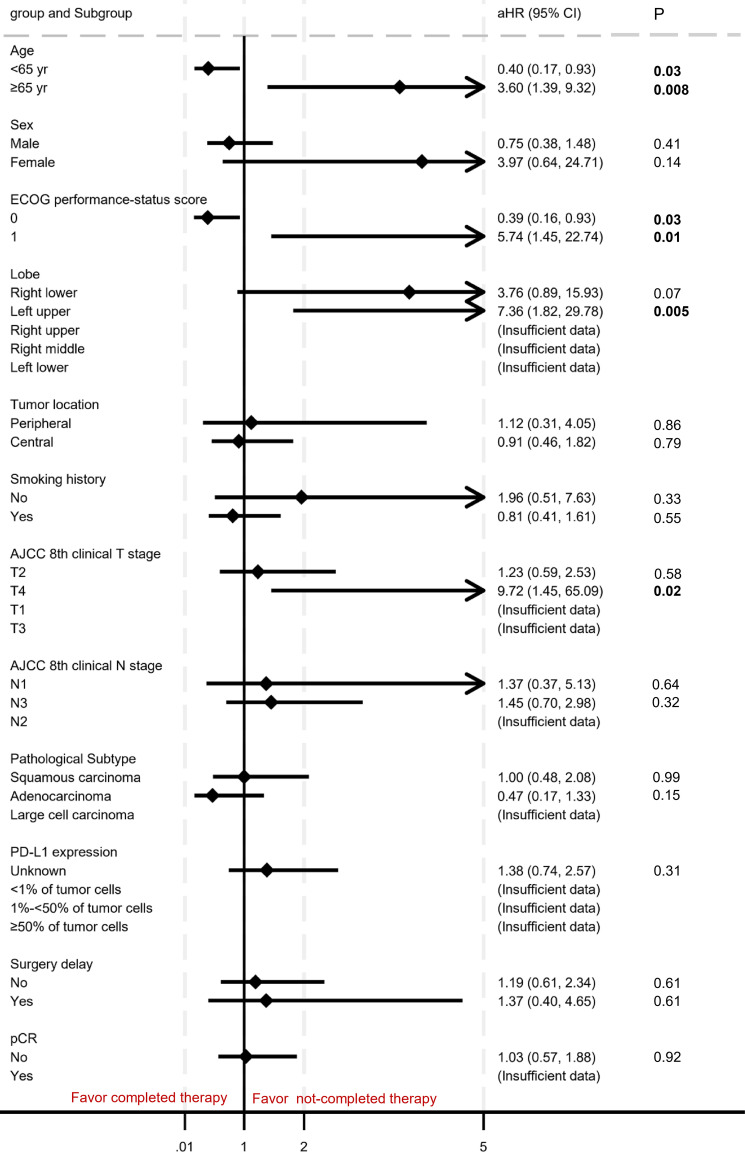
Subgroup analysis of recurrence-free survival between completing full standard of therapy and not-completed therapy for resectable non-small cell lung cancer.

Subgroup analysis was performed to identify patient populations that might derive differential benefit from completing perioperative chemoimmunotherapy (**Figure 3**). In the IPTW-adjusted analysis, patients aged <65 years (aHR 0.40, 95% CI 0.17-0.93, P = 0.03) and ECOG performance-status score=0 (aHR 0.39, 95% CI 0.16-0.93, P = 0.03), appeared to derive RFS benefit from completing the full regimen.

## Discussion

The introduction of ICIs has reshaped the treatment landscape for resectable NSCLC. Crucial Phase 3 trials have firmly established neoadjuvant (Checkmate 816) and perioperative chemoimmunotherapy (Checkmate 77T, AEGEAN, and Keynote 671), demonstrating significant improvements in pathological remission, EFS, and, notably, OS in Keynote 671 (5–8). These findings have led major international guidelines to recommend perioperative immunotherapy-based approaches for eligible patients. The NCCN Guidelines (17) recommend neoadjuvant chemoimmunotherapy (3–4 cycles) followed by surgery and one year of adjuvant single-agent immunotherapy for eligible patients. The ESMO Clinical Practice Guidelines (10) and IASLC consensus (11) recommendations similarly endorse perioperative chemoimmunotherapy as a standard of care. However, the high rates of non-adherence, particularly with the prolonged 12-month adjuvant component posed a significant challenge to translating these theoretical benefits into real-world practice. Prior studies reported completion rates for the adjuvant immunotherapy as low as 24% in Keynote 671 (8). Consequently, a critical question for practicing clinicians is whether the failure to adhere to this demanding, long-term protocol negatively impacts patient survival, and which patient subgroups are most susceptible to this risk. Our retrospective, real-world study sought to address this gap by comparing long-term outcomes of patients who completed the full standard regimen against those who did not, using IPTW to minimize confounding bias. It is important to note that the two post-treatment variables (ypN stage and pCR), which were not included in the IPTW model, showed differences after IPTW. This is expected because these are treatment outcomes rather than baseline confounders, and their post-IPTW differences reflect the association between treatment completion and pathological response rather than residual confounding.

IPTW-adjusted analyses revealed no significant differences in RFS or OS between the completed and non-completed groups. This finding, while unexpected given the established trial benefits, underscores the challenges of translating controlled efficacy into real-world effectiveness. Notably, direct cross-trial comparison of absolute outcomes between CheckMate 816 (neoadjuvant-only) and CheckMate 77T (perioperative) is inappropriate due to differences in eligibility criteria, and treatment schemas. However, indirect comparison using hazard ratios suggests comparable oncological benefit, the pCR rate was 24.0% in Checkmate 816 (odds ratio [OR] 13.94, 99% CI 3.49-55.75, P<0.001) and was 25.3% in Checkmate 77T (OR 6.64, 99% CI 3.40-12.97). Meanwhile, the median EFS of the former group was 31.6 months (HR 0.63, 97.38% CI 0.43-0.91, P = 0.005), while the latter group is expected to be over 30 months (HR 0.58, 97.36% CI 0.42-0.81, P < 0.001), and there does not seem to be a significant difference either (5, 6). An indirect meta-analysis suggested that adding adjuvant immunotherapy to neoadjuvant chemoimmunotherapy did not improve survival outcomes for patients with resectable NSCLC (18). Some retrospective studies have inconsistencies.

Recent evidence has highlighted that adjuvant immunotherapy benefit may differ substantially based on pathological response status. However, the benefit for non-pCR patients remains controversial. A study reported no improvement with adjuvant immunotherapy regardless of pCR status in stage III NSCLC (19).Another study suggested that EFS was significantly prolonged with adjuvant immunotherapy in stage IB-IIB patients who did not achieve a pCR (20). In our cohort, pCR was not an independent predictor of RFS (aHR 0.68, 95% CI 0.10-4.67, P = 0.70), suggesting its apparent protective effect in univariate analysis was largely mediated by pathological downstaging (ypT and ypN stage). This finding reaffirms that achieving favorable pathological nodal status (ypN0) remains the most critical factor for long-term prognosis, regardless of completion status.

IPTW-adjusted multivariate Cox regression reaffirmed the dominant prognostic value of post-treatment pathological staging. Positive ypN status was an independent predictor of recurrence (ypN2: aHR 35.56, 95% CI 10.71-118.08), whereas completion of perioperative therapy was not. For patients with positive ypN status, adjuvant therapy remains necessary to reduce recurrence risk, though the wide confidence interval warrants cautious interpretation. We acknowledge that access to adjuvant immunotherapy varies globally. Some regions, particularly in low- and middle-income countries, still lack reimbursement for adjuvant immunotherapy altogether. Regarding safety, the completed group experienced higher incidence of any-grade myelosuppression and irAEs, but no significant increase in Grade 3–4 AEs, consistent with a meta-analysis of multiple RCTs showing increased any-grade toxicity without significantly elevated severe toxicity (18).

Subgroup analysis revealed that patients aged <65 years and those with ECOG performance-status score of 0 derived greater RFS benefit from completing perioperative immunotherapy. Younger patients may have more robust immune systems with greater capacity for immune reconstitution during prolonged immunotherapy, allowing them to better tolerate the full treatment course. Ptients with ECOG performance-status score = 0 have fully preserved activity levels and better overall physiological reserve and immune function compared with those with PS = 1. These patients are more likely to tolerate the cumulative toxicity of a complete perioperative regimen without dose reductions or treatment interruptions, thereby receiving the full therapeutic intensity required for optimal benefit. In an indirect meta-analysis comparing neoadjuvant chemoimmunotherapy plus adjuvant immunotherapy with neoadjuvant chemoimmunotherapy only, subgroup analysis did not reveal a significant tendency towards completion of the treatment in any subgroups of age, sex, pathological subtype, smoking status, pCR, and PD-L1 expression (18). Therefore, we should be cautious when interpreting the results of subgroup analysis. Circulating tumor DNA (ctDNA) may help identify which patients truly require complete adjuvant immunotherapy versus those who may safely discontinue treatment. In the CheckMate-816 trial, ctDNA clearance after neoadjuvant therapy was significantly correlated with pCR, and ctDNA clearance was associated with improved event-free survival regardless of pCR status (21). Similarly, the AEGEAN study demonstrated that patients achieving ctDNA clearance after neoadjuvant therapy had significantly better EFS outcomes (22). Trials such as ADOPT-LUNG are evaluating ctDNA-guided treatment de-escalation strategies (23). However, current ctDNA assays still have insufficient sensitivity for minimal residual disease detection in early-stage NSCLC, and standardized platforms are needed before widespread clinical adoption (24).

This study has several limitations. First, retrospective design introduces potential selection and information bias; while IPTW balanced measured covariates, unmeasured confounding cannot be entirely eliminated. Second, the relatively small completed group reduces statistical power and increases the risk of type II errors. Incomplete PD-L1 data further precluded definitive subgroup analysis. Third, single-center experience limits generalizability, warranting multicenter external validation. Fourth, treatment dropout and alternative treatments, may have introduced selection bias, as these higher-risk patients were excluded. Fifth, our data cannot independently adjudicate the relative contributions of neoadjuvant cycle number versus adjuvant therapy duration. Finally, the median follow-up of 20 months is relatively short, and survival differences may emerge with longer observation. Our results should therefore be interpreted with caution.

## Conclusion

Despite these limitations, our real-world study offers meaningful insights into perioperative immunotherapy implementation. Our data demonstrate that completing the full protocol is challenging and is not associated with a survival benefit in the overall population. The decision to pursue complete perioperative chemoimmunotherapy should be personalized, incorporating robust prognostic factors such as age and performance status. Future efforts should focus on validating predictive biomarkers to better identify patients who truly require and will benefit from the complete perioperative treatment strategy.

## Data Availability

The datasets presented in this article are not readily available because of privacy and ethical restrictions. Requests to access the datasets should be directed to the corresponding author.

## Supplementary material

Supplementary Figure 1 Flowchart of selection of study cohort. CJFH, China-Japan Friendship Hospital; SCLC, small cell lung cancer; NSCLC, non-small cell lung cancer; ECOG, eastern cooperative oncology group.

Supplementary Figure 2 Love plot: covariate balance improvement after IPTW. Green zone, SMD = 0.1 (well−balanced); Arrows show change after weighting. COPD, Chronic obstructive pulmonary disease; ECOG, eastern cooperative oncology group; DLCO SB, Diffusing capacity of the lung for carbon monoxide, single−breath method; %, Its percentage of prediction value; FVC, Forced−vital capacity; FEV1, Forced expiratory volume in 1 s; FEV1/FVC, The percentage calculated by dividing FEV1 by FVC; PD-L1, programmed death ligand 1.

Supplementary Figure 3 IPTW sensitivity analysis - E-values. Larger E-values indicate more robust results against unmeasured confounding; Red dashed, E = 1.25 (sensitive); Orange dashed, E = 1.5 (moderate); Green dashed, E = 2.0 (robust). MAE, Myelosuppression adverse event; AE, adverse event; irAEs, immune-related adverse event; G3, grade≥3.

Supplementary Figure 4 Sankey diagram - an overview of treatments which patients received in the study. 4 cycles NCI, Completed 4 cycles of neoadjuvant chemoimmunotherapy; < 4 cycles NCI, Completed < 4 cycles of neoadjuvant chemoimmunotherapy; Lacked cycles CI completed, Completed lacked cycles of chemoimmunotherapy after surgery; Lacked cycles CI Not completed, Not completed lacked cycles of chemoimmunotherapy after surgery; 1 year AI completed, Completed one year of adjuvant Immunotherapy; 1 year AI Not completed, Not completed one year of adjuvant immunotherapy.
