# Supplementary material for: Is full adherence mandatory? Real-world outcomes of completing perioperative chemoimmunotherapy in resectable non-small cell lung cancer
Source: Front Oncol. 2026 May 28;16:1837880. doi: 10.3389/fonc.2026.1837880 (PMC13253235; doi:10.3389/fonc.2026.1837880)
Supplement: Supplementary Figure 1 — Flowchart of selection of study cohort. CJFH, China-Japan Friendship Hospital; SCLC, small cell lung cancer; NSCLC, non-small cell lung cancer; ECOG, eastern cooperative oncology group. [file Image1.pdf]

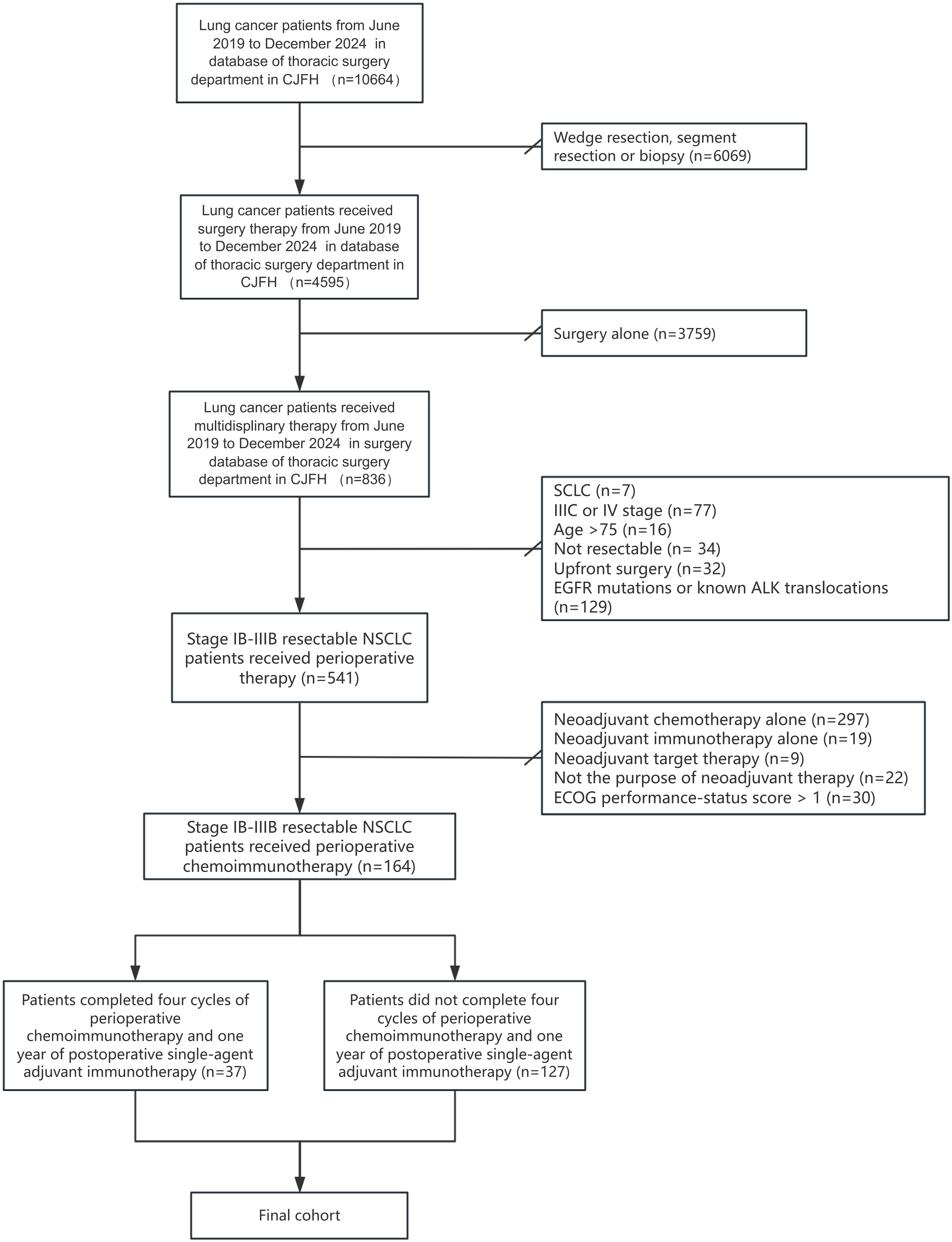
Lung cancer patients from June 2019 to December 2024 in database of thoracic surgery department in CJFH （n=10664）
Wedge resection, segment resection or biopsy (n=6069)
Lung cancer patients received surgery therapy from June 2019 to December 2024 in database of thoracic surgery department in CJFH （n=4595）
Surgery alone (n=3759)
Lung cancer patients received multidisplinary therapy from June 2019 to December 2024 in surgery database of thoracic surgery department in CJFH （n=836）
SCLC (n=7)
IIIC or IV stage (n=77)
Age >75 (n=16)
Not resectable (n= 34)
Upfront surgery (n=32)
EGFR mutations or known ALK translocations (n=129)
Stage IB-IIIB resectable NSCLC patients received perioperative therapy (n=541)
Neoadjuvant chemotherapy alone (n=297)
Neoadjuvant immunotherapy alone (n=19)
Neoadjuvant target therapy (n=9)
Not the purpose of neoadjuvant therapy (n=22)
ECOG performance-status score > 1 (n=30)
Stage IB-IIIB resectable NSCLC patients received perioperative chemoimmunotherapy (n=164)
Patients completed four cycles of perioperative chemoimmunotherapy and one year of postoperative single-agent adjuvant immunotherapy (n=37)
Patients did not complete four cycles of perioperative chemoimmunotherapy and one year of postoperative single-agent adjuvant immunotherapy (n=127)
Final cohort
