# Supplementary figures and images for: Is full adherence mandatory? Real-world outcomes of completing perioperative chemoimmunotherapy in resectable non-small cell lung cancer
Source: Front Oncol. 2026 May 28;16:1837880. doi: 10.3389/fonc.2026.1837880 (PMC13253235; doi:10.3389/fonc.2026.1837880)

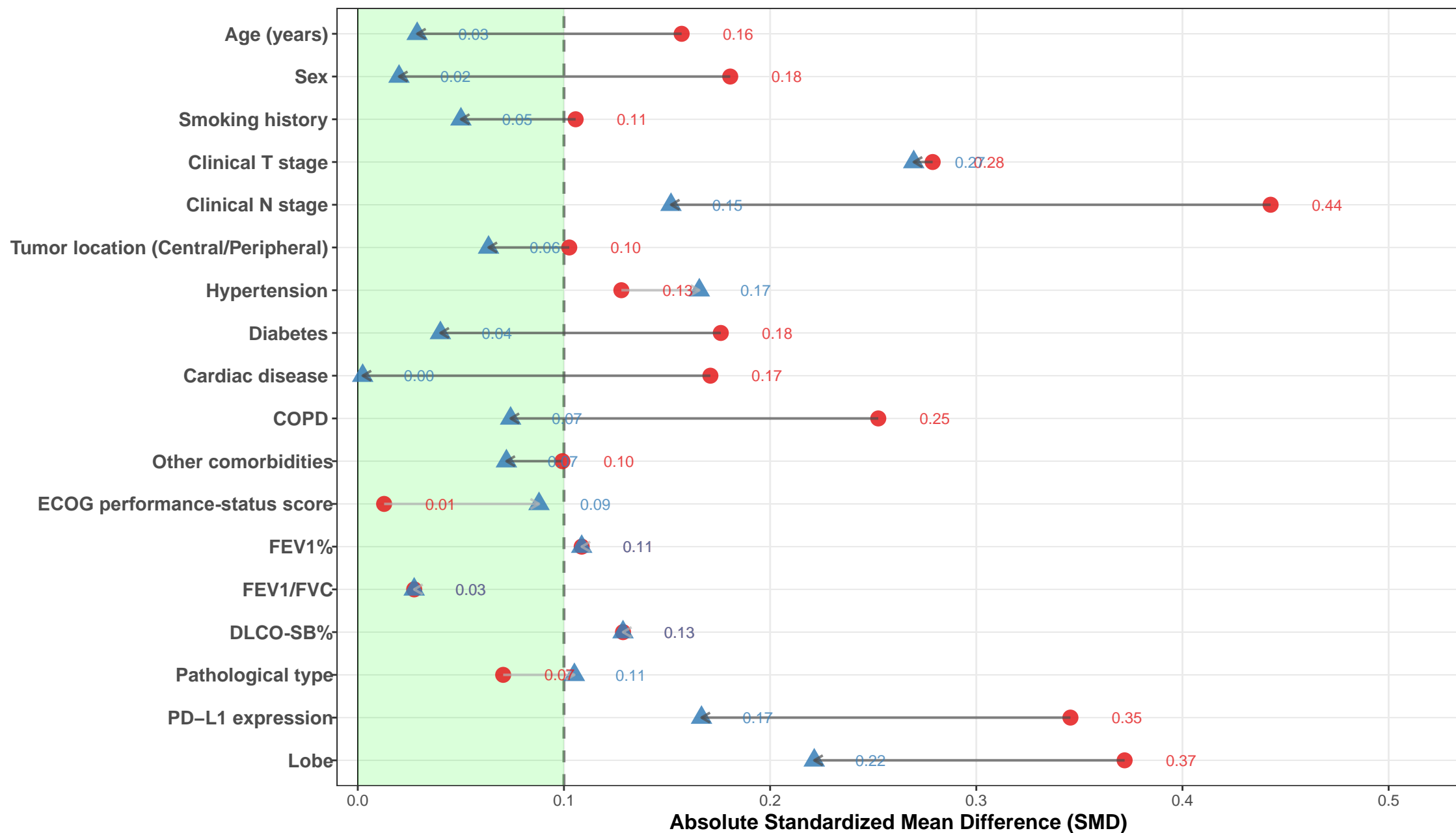

Group → . No change → . Improved ● After IPTW ● Before IPTW

Supplement: Supplementary Figure 2 — Love plot: covariate balance improvement after IPTW. Green zone, SMD = 0.1 (well−balanced); Arrows show change after weighting. COPD, Chronic obstructive pulmonary disease; ECOG, eastern cooperative oncology group; DLCO SB, Diffusing capacity of the lung for carbon monoxide, single−breath method; %, Its percentage of prediction value; FVC, Forced−vital capacity; FEV1, Forced expiratory volume in 1 s; FEV1/FVC, The percentage calculated by dividing FEV1 by FVC; PD-L1, programmed death ligand 1. [file Image2.pdf]

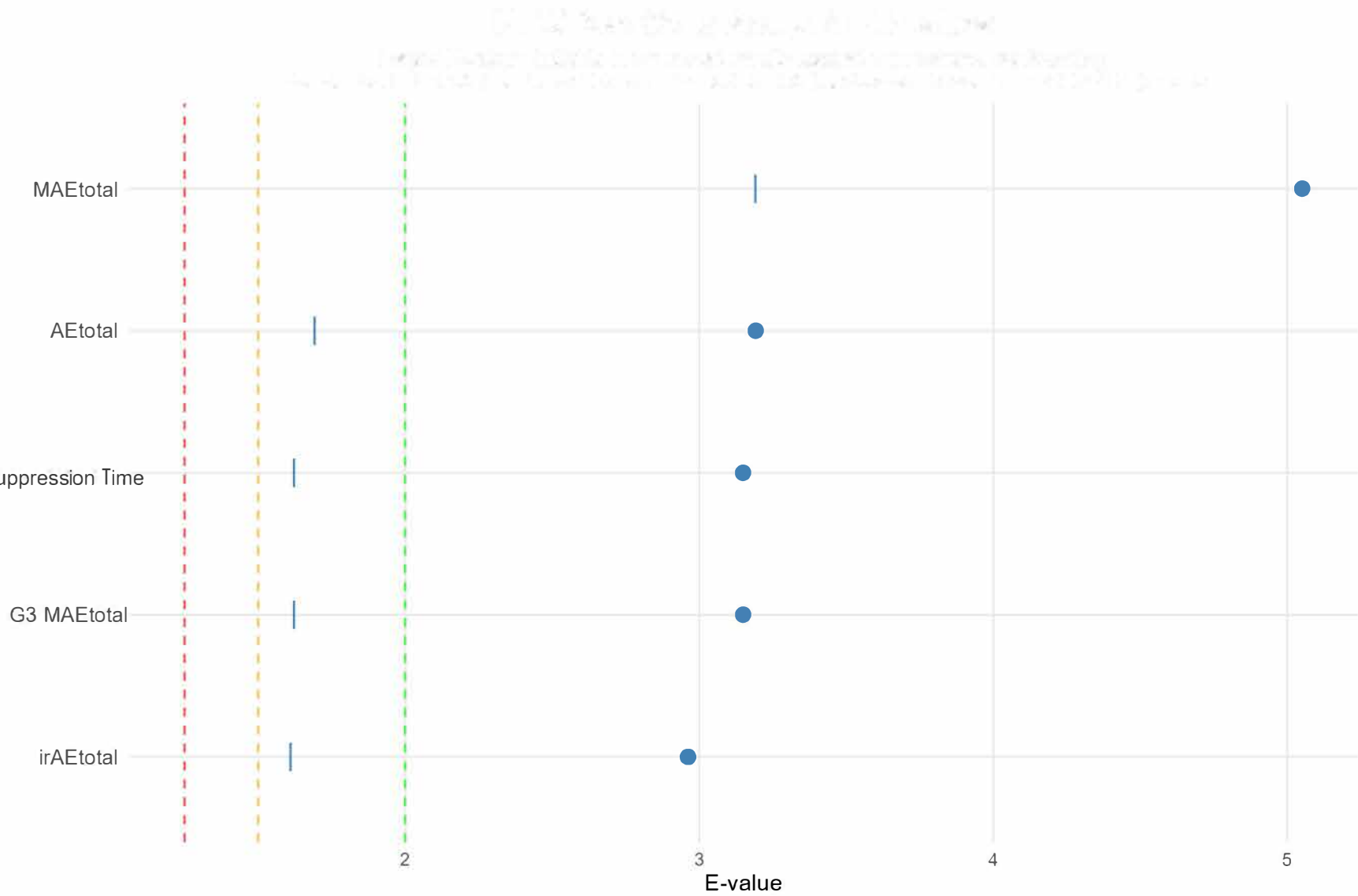

Supplement: Supplementary Figure 3 — IPTW sensitivity analysis - E-values. Larger E-values indicate more robust results against unmeasured confounding; Red dashed, E = 1.25 (sensitive); Orange dashed, E = 1.5 (moderate); Green dashed, E = 2.0 (robust). MAE, Myelosuppression adverse event; AE, adverse event; irAEs, immune-related adverse event; G3, grade≥3. [file Image3.pdf]

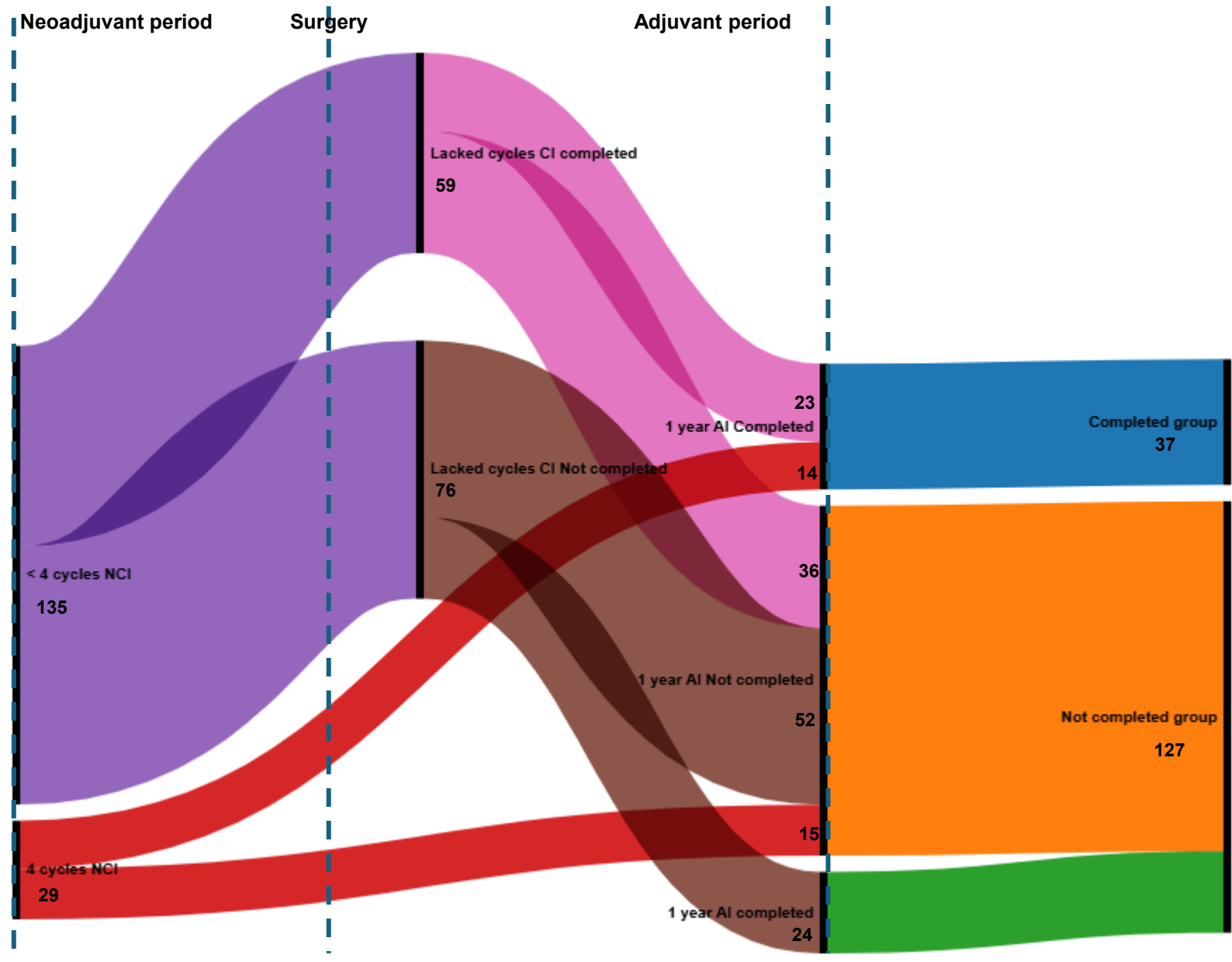

Supplement: Supplementary Figure 4 — Sankey diagram - an overview of treatments which patients received in the study. 4 cycles NCI, Completed 4 cycles of neoadjuvant chemoimmunotherapy; < 4 cycles NCI, Completed < 4 cycles of neoadjuvant chemoimmunotherapy; Lacked cycles CI completed, Completed lacked cycles of chemoimmunotherapy after surgery; Lacked cycles CI Not completed, Not completed lacked cycles of chemoimmunotherapy after surgery; 1 year AI completed, Completed one year of adjuvant Immunotherapy; 1 year AI Not completed, Not completed one year of adjuvant immunotherapy. [file Image4.pdf]
