# Supplementary material for: Is full adherence mandatory? Real-world outcomes of completing perioperative chemoimmunotherapy in resectable non-small cell lung cancer
Source: Front Oncol. 2026 May 28;16:1837880. doi: 10.3389/fonc.2026.1837880 (PMC13253235; doi:10.3389/fonc.2026.1837880)
Supplement: Supplementary file 5 [file Table1.docx]

Table S1 The regimens for chemotherapy and immunotherapy

|  | Total cohort (n=164) | Completed group (n=37) | Not-completed group (n=127) | P |
| --- | --- | --- | --- | --- |
| Chemotherapy regimen, n(%) |  |  |  | 0.51 |
| Paclitaxel+platinum | 133(81.1) | 32(86.5) | 101(79.5) |  |
| Pemetrexed+platinum | 24(14.6) | 3(8.1) | 21(16.5) |  |
| Gemcitabine+platinum | 7(4.3) | 2(5.4) | 5(3.9) |  |
| Immunotherapy regimen, n(%) |  |  |  | 0.06 |
| Durvalumab | 7(4.3) | 4(10.8) | 3(2.4) |  |
| Camrelizumab | 4(2.4) | 1(2.7) | 3(2.4) |  |
| Nivolumab | 4(2.4) | 2(5.4) | 2(1.6) |  |
| Pembrolizumab | 34(20.7) | 9(24.3) | 25(19.7) |  |
| Toripalimab | 17(10.4) | 5(13.5) | 12(9.4) |  |
| Tislelizumab | 74(45.1) | 10(27) | 64(50.4) |  |
| Sintilimab | 24(14.6) | 6(16.2) | 18(14.2) |  |
