# Supplementary material for: Is full adherence mandatory? Real-world outcomes of completing perioperative chemoimmunotherapy in resectable non-small cell lung cancer
Source: Front Oncol. 2026 May 28;16:1837880. doi: 10.3389/fonc.2026.1837880 (PMC13253235; doi:10.3389/fonc.2026.1837880)
Supplement: Supplementary file 6 [file Table2.docx]

Table S2 Perioperative outcomes

| Variables | Completed group (n=37) | Not-completed group (n=127) | P |
| --- | --- | --- | --- |
| Surgery approach, n(%) |  |  | 0.16 |
| VATS | 19(51.4) | 77(60.6) |  |
| Conversion | 10(27) | 38(29.9) |  |
| Thoracotomy | 8(21.6) | 12(9.4) |  |
| Operation procedure, n(%) |  |  | 0.31 |
| Lobectomy | 25(67.6) | 87(68.5) |  |
| Bilobectomy | 4(10.8) | 24(18.9) |  |
| Sleeve Lobectomy | 5(13.5) | 12(9.4) |  |
| Pneumonectomy | 3(8.1) | 4(3.1) |  |
| Operation duration(min), median(Q1,Q3) | 164.5(137.5,202.5) | 159(120,190) | 0.43 |
| Bleeding volume(ml), median(Q1,Q3) | 50(20,100) | 50(20,100) | 0.26 |
| Resection completeness, n(%) |  |  | 1 |
| R0 | 35(94.6) | 120(94.5) |  |
| R1/2 | 2(5.4) | 7(5.5) |  |
| Chest tube duration(days), median(Q1,Q3) | 5(4,6) | 5(4,6) | 0.87 |
| Postoperative hospital duration(days), median(Q1,Q3) | 6(5,7) | 6(5,7) | 0.97 |
| First day drainage volume(ml), median(Q1,Q3) | 250(150,300) | 280(150,367) | 0.40 |
| Postoperative complications, n(%) |  |  |  |
| Pneumothorax | 0 | 8(6.3) | 0.20 |
| Hemothorax | 1(2.7) | 1(0.8) | 0.40 |
| Subcutaneous emphysema | 0 | 3(2.4) | 1 |
| Pulmonary infection | 6(16.2) | 21(16.5) | 1 |
| Pulmonary atelectasis | 3(8.1) | 6(4.7) | 0.42 |
| Pulmonary embolism | 1(2.7) | 0 | 0.23 |
| Prolonged air leak | 1(2.7) | 3(2.4) | 1 |
| Pleural effusion | 2(5.4) | 8(6.3) | 1 |
| Atrial fibrillation | 2(5.4) | 7(5.5) | 1 |
| Heart failure | 0 | 0 | 1 |
| Cerebral infarction | 1(2.7) | 2(1.6) | 0.54 |

VATS: Video-assisted Thoracoscopic Surgery, Q1: first percentile, Q3: third percentile.
