# Supplementary material for: Is full adherence mandatory? Real-world outcomes of completing perioperative chemoimmunotherapy in resectable non-small cell lung cancer
Source: Front Oncol. 2026 May 28;16:1837880. doi: 10.3389/fonc.2026.1837880 (PMC13253235; doi:10.3389/fonc.2026.1837880)
Supplement: Supplementary file 8 [file Table4.docx]

Table S4 Adverse events details in adjuvant period

| Variables | Completed group (n=37) | | | |  | Not-completed group (n=127) | | | |
| --- | --- | --- | --- | --- | --- | --- | --- | --- | --- |
|  | G1 | G2 | G3 | G4 |  | G1 | G2 | G3 | G4 |
| AE related to myelosuppression, n(%) |  |  |  |  |  |  |  |  |  |
| Leukopenia | 1(2.7) | 0 | 0 | 2(5.4) |  | 3(2.4) | 3(2.4) | 3(2.4) | 0 |
| Neutropenia | 2(5.4) | 0 | 0 | 1(2.7) |  | 2(1.6) | 2(1.6) | 1(0.8) | 4(3.1) |
| Anemia | 1(2.7) | 2(5.4) | 0 | 0 |  | 3(2.4) | 2(1.6) | 2(1.6) | 0 |
| Thrombocytopenia | 3(8.1) | 0 | 1(2.7) | 0 |  | 2(1.6) | 0 | 2(1.6) | 0 |
| irAE, n(%) |  |  |  |  |  |  |  |  |  |
| Colitis | 1(2.7) | 0 | 0 | 0 |  | 0 | 1(0.8) | 1(0.8) | 0 |
| Hepatitis | 1(2.7) | 0 | 1(2.7) | 0 |  | 0 | 0 | 2(1.6) | 0 |
| Pneumonitis | 1(2.7) | 0 | 0 | 0 |  | 1(0.8) | 2(1.6) | 0 | 0 |
| Myocarditis | 1(2.7) | 0 | 0 | 0 |  | 1(0.8) | 0 | 0 | 1(0.8) |
| Hyperthyroidism/Hypothyroidism | 3(8.1) | 0 | 0 | 0 |  | 0 | 1(0.8) | 0 | 0 |
| Nephritis | 1(2.7) | 0 | 0 | 0 |  | 1(0.8) | 0 | 0 | 0 |
| Erythra | 2(5.4) | 0 | 0 | 0 |  | 2(1.6) | 1(0.8) | 1(0.8) | 0 |
| Diabetes | 0 | 0 | 0 | 0 |  | 1(0.8) | 0 | 0 | 0 |
| Hypoadrenocorticism | 3(8.1) | 2(5.4) | 0 | 0 |  | 1(0.8) | 0 | 0 | 0 |

AE: adverse event; irAE: immune−related adverse event; G: grade
