# Supplementary material for: Is full adherence mandatory? Real-world outcomes of completing perioperative chemoimmunotherapy in resectable non-small cell lung cancer
Source: Front Oncol. 2026 May 28;16:1837880. doi: 10.3389/fonc.2026.1837880 (PMC13253235; doi:10.3389/fonc.2026.1837880)
Supplement: Supplementary file 9 [file Table5.docx]

Table S5 Number of cycles received in two groups

| Variables | Completed group (n=37) | Not-completed group (n=127) | P |
| --- | --- | --- | --- |
| Number of therapy cycles in neoadjuvant period, n(%) | | | **<0.001** |
| 2 | 6(16.2) | 69(54.3) |  |
| 3 | 18(48.7) | 42(33.1) |  |
| 4 | 13(35.1) | 16(12.6) |  |
| Number of therapy cycles in adjuvant period, median(Q1,Q3) | 13(11,16) | 2(0,4) | **<0.001** |

Q1: first percentile, Q3: third percentile.
